# Decreased expression of PBLD correlates with poor prognosis and functions as a tumor suppressor in human hepatocellular carcinoma

**DOI:** 10.18632/oncotarget.6358

**Published:** 2015-11-22

**Authors:** Aimin Li, Qun Yan, Xinmei Zhao, Jietao Zhong, Haiyun Yang, Zhiqiang Feng, Yanlei Du, Yadong Wang, Zenan Wang, Hong Wang, Yongjian Zhou, Side Liu, Yuqiang Nie

**Affiliations:** ^1^ Department of Gastroenterology, Guangdong Provincial Key Laboratory of Gastroenterology, Nanfang Hospital, Southern Medical University, Guangzhou, China; ^2^ Department of Gastroenterology, Guangzhou Key Laboratory of Digestive Disease, Guangzhou First Municipal People's Hospital, Guangzhou Medical University, Guangzhou, China; ^3^ Division of Allergy and Clinical Immunology, The Johns Hopkins School of Medicine, Baltimore, MD, United States of America; ^4^ Department of Physiology, Yong Loo Lin School of Medicine, National University of Singapore, Singapore, Singapore

**Keywords:** hepatocellular carcinoma, tumorgenesis, PBLD, tumor suppressor gene, prognosis

## Abstract

Recent accumulating genomic and proteomic data suggested that decreased expression of phenazine biosynthesis-like domain-containing protein (PBLD) was frequently involved in hepatocellular carcinoma (HCC). However, there is lack of systematical investigation focusing on its expression pattern, clinical relevance, and biological function. Here, we found that PBLD was frequently decreased in HCC tissues relative to adjacent non-tumorigenic liver tissues. This decreased expression was significantly associated with poor tumor differentiation and advanced tumor stage. Kaplan–Meier analysis further showed that recurrence-free survival and overall survival were significantly worse among patients with low PBLD expression. Moreover, multivariate analyses revealed that PBLD was an independent predictor of OS and RFS. This prognostic value of PBLD was further validated in another independent cohort. We also found PBLD inhibited HCC cell growth and invasion *in vitro* and tumor growth in *vivo*. Furthermore, forced expression of PBLD influenced multiple downstream genes related to MAPK, NF-κB, EMT, and angiogenesis signaling pathways. PBLD deletion was an independent predictor of poor prognosis in patients with HCC. Elevated PBLD expression may reduce HCC cell growth and invasion via inactivation of several tumorigenesis-related signaling pathways.

## INTRODUCTION

Hepatocellular carcinoma (HCC) ranks as the sixth most common cancer and the third leading cause of cancer-related mortality worldwide [1], accounting for approximately 75%-90% of malignant tumors in adult livers [2]. The prognosis of HCC is poor because the disease is often at a fairly advanced stage at the time of diagnosis. Surgical treatments including liver resection and liver transplantation are effective therapies for patients with early HCC at present. However, the clinical outcome is still unsatisfactory due to its high recurrence rate after surgery [3]. Although more and more molecular markers with high sensitivity and specificity for HCC have been proposed, none has justified its routine use in clinical practice till now [4, 5]. Hence, a better understanding of more molecular markers and pathways that contribute to the progression and recurrence of HCC is essential for the development of more effective, targeted therapies.

As powerful molecular techniques at present, gene expression profiling and proteomics analysis have been extensively used to improve the identification of new biomarkers leading to early diagnosis and more effective therapies, and the definition of the mechanisms associated with HCC progression [6]. Using gene expression microarray analysis, molecular features of non-B and non-C HCC (HCC negative for both HBV and HCV) [7], HBV-associated HCC [8, 9] and HCV-associated HCC [9] were characterized and the differential gene were screened compared with their corresponding non-tumorous liver tissues in three independent experiments. Phenazine biosynthesis-like domain-containing protein (PBLD) was common to all three studies and the mRNA level of PBLD expression was observed to decrease significantly in HCC tumors compared with that in paired non-tumorous tissues. Consistently, proteomics analysis revealed that the protein level of PBLD expression was also dramatically decreased in HCC tissues [10]. In addition, PBLD was significantly downregulated in HCC tissues with portal vein tumor thrombus (PVTT) and HCC recurrence compared with that without PVTT and recurrence in genomic and proteomic analyses [11, 12]. These findings suggested a potential role of PBLD in the carcinogenesis and progression of liver cancer.

PBLD, also termed as MAWBP, was first identified from a human liver cDNA library using a yeast two-hybrid technique by Iriyama *et al.* [13]. As the only representative of phenazine biosynthesis-like protein family, PBLD is widely expressed in human tissues, such as brain, heart, lung, liver, pancreas, kidney, and placenta. Its expression is elevated in several diseases processes, including insulin resistance, folate deficiency, and hypotension. More recently, PBLD has been proved to be involved in gastric carcinogenesis. Zhang *et al.* [14] found that PBLD expression was attenuated in gastric cancer (GC) tissues. Li *et al.* [15] further confirmed that PBLD negatively regulated the growth and invasion in gastric cancer cell-lines by inhibiting TGF-β1-induced EMT (Epithelial-Mesenchymal Transition). Until now, however, there is lack of the systematical investigation focusing on its expression pattern, clinical relevance, and biological function in HCC. The limited reports were mostly achieved from the screening experiments of genomics and proteomic survey as described above.

In the present study, we investigated the expression of PBLD in HCC tissues after curative resection by quantitative real-time PCR, immunohistochemistry, and western blotting. As the results show, PBLD was frequently decreased in HCC tissues and this downregulation was significantly associated with poor prognosis of HCC patients. We also analyzed *in vitro* and *in vivo* the role of PBLD overexpression in inhibiting HCC cell growth and metastasis. Furthermore, using microarray analysis, we found that the antitumorigenic effects of PBLD overexpression were likely associated with the inhibition of multiple tumor progression–related signaling pathways, including vascular endothelial growth factor-A (VEGF-A), mitogen-activated protein kinase (MAPK), nuclear factor κB (NF-κB), epithelial-mesenchymal transition (EMT), angiogenesis and others, even if the precise mechanism of PBLD for tumor inhibition is yet not entirely clear and requires further study.

## RESULTS

### PBLD is downregulated in HCC

**Figure 1:**
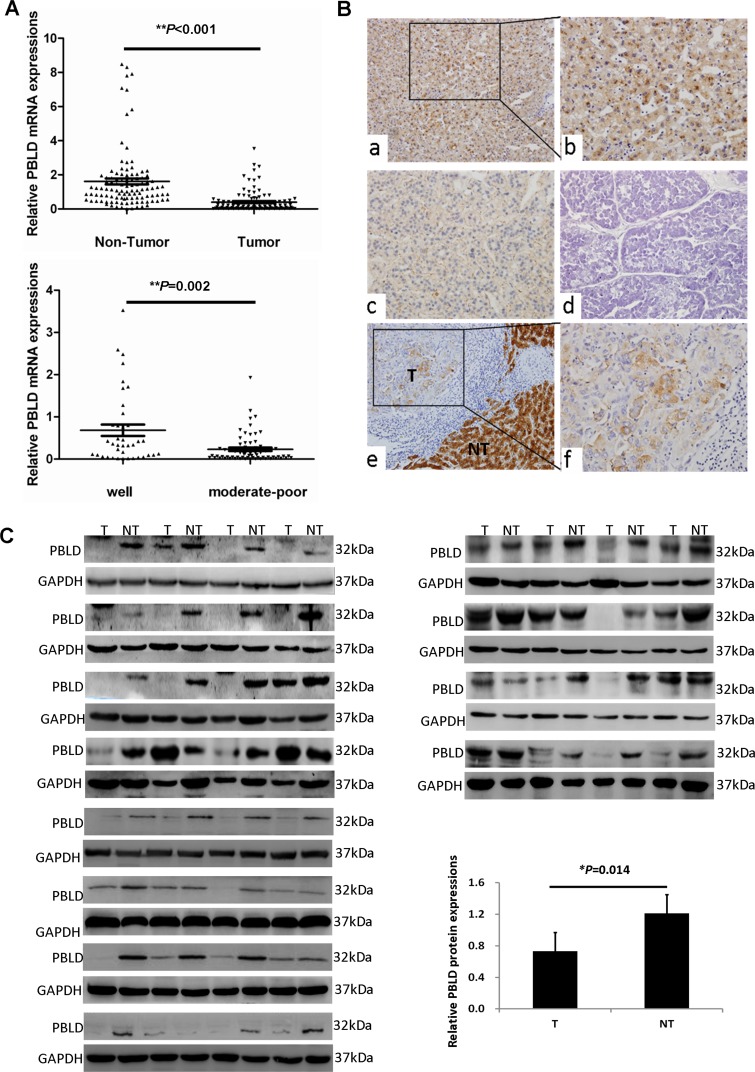
PBLD is aberrantly downregulated in human HCCs **A**. The relative mRNA expression of PBLD in HCC tissues was decreased when compared with the matched adjacent non-tumorigenic tissues by qRT-PCR (n = 108, *P* < 0.001) (top). Additionally, PBLD expression was markedly higher in well-differentiated HCC tissues as compared with moderate to poorly-differentiated HCC tissues (bottom). **B**. Representative immunohistochemical staining for PBLD in adjacent non-tumorous liver tissues (a, ×200; b, ×400), and in well (c, ×200), moderately (f, ×400), and poorly (d, ×200) differentiated HCC tissues. The staining for PBLD was dramatically decreased in HCC tissues as compared with that shown for non-tumorous liver tissues, which showed a sharp contrast between the tumorous area (“T”) and the adjacent non-tumorous area (“NT”) (e, ×200). **C**. PBLD protein expression was detected in the 48 paired of HCC and matched adjacent non-tumorous tissues as determined by western blotting.

We first examined the frequency of PBLD mRNA expression by qRT-PCR on larger sample sets. Notably, as shown in Figure 1A, PBLD mRNA expression was significantly decreased in HCC tissues compared with that in adjacent non-tumorous tissues, which was detected in 84.3% (91/108) of HCC patients, and PBLD mRNA expression in moderate to poor-differentiated HCC tissues was more frequently downregulated than that in well-differentiated tissues (*P* = 0.002).

To further confirm the expression of PBLD, we carried out Western blotting and immunohistochemical analyses with 48 pairs of HCC and adjacent non-tumorous tissues, and its expression was also significantly decreased in HCC tissues at protein levels relative to non-tumorous tissues (*P* < 0.05). With regard to the localization of PBLD protein, it was predominantly detected in the cytoplasm of hepatic cells with a little staining in the nuclei by immunohistochemistry. Surrounding non-tumorous liver tissues displayed extensively positive staining for PBLD, whereas HCC tissues showed variable proportions of PBLD-positive cells that gradually decreased with the grades of differentiation from well differentiated to poorly differentiated (Figure 1B). Meanwhile, PBLD expression was decreased in 41 of 48 cases of HCC tissues as compared with matched adjacent non-tumorous tissues by western blot analysis (Figure 1C). These results indicate that PBLD is aberrantly downregulated in a subset of human HCCs.

### Decreased PBLD expression predicts poor prognosis in HCC patients

To investigate the significance of PBLD expression in HCC, PBLD expression levels detected by qRT-PCR were correlated with specific clinicopathologic features of 108 patients with HCC. As shown in Table 1, high PBLD expression was closely correlated with tumor differentiation (*P* = 0.017) and tumor stage (*P* = 0.038). However, the expression of PBLD was not significantly correlated with age, gender, hepatitis B surface antigen (HBsAg), hepatitis B e antigen (HBeAg), serum AFP level, liver cirrhosis, vascular invasion, intrahepatic metastasis, tumor number and tumor size.

**Table 1:** The association of PBLD mRNA expression with the clinicopathological characteristics in 108 patients with HCC

Variables	n	PBLD		*P*
		Low (n = 54)	High (n = 54)	
Age (years)				
<50	62	34	28	0.243
≥50	46	20	26	
Gender				
Male	97	50	47	0.340
Female	11	4	7	
HBsAg				
positive	99	48	51	0.296
negative	9	6	3	
HBeAg				
positive	14	7	7	1.000
negative	94	47	47	
AFP* (ng/mL)				
>400	60	34	26	0.121
≤400	48	20	28	
Liver Cirrhosis				
Absent	30	19	11	0.086
Present	78	35	43	
Vascular invasion				
Absent	83	40	44	0.494
Present	25	14	10	
Intrahepatic metastasis				
Absent	83	38	45	0.110
Present	25	16	9	
Tumor size				
≤5 cm	25	11	14	0.494
>5 cm	83	43	40	
Tumor number				
Single	81	37	44	0.120
Multiple	27	17	10	
Tumor differentiation				
Well	40	14	26	0.017
Moderate-Poor	68	40	28	
AJCC stage				
I/II	74	32	42	0.038
III/IV	34	22	12	

**Figure 2:**
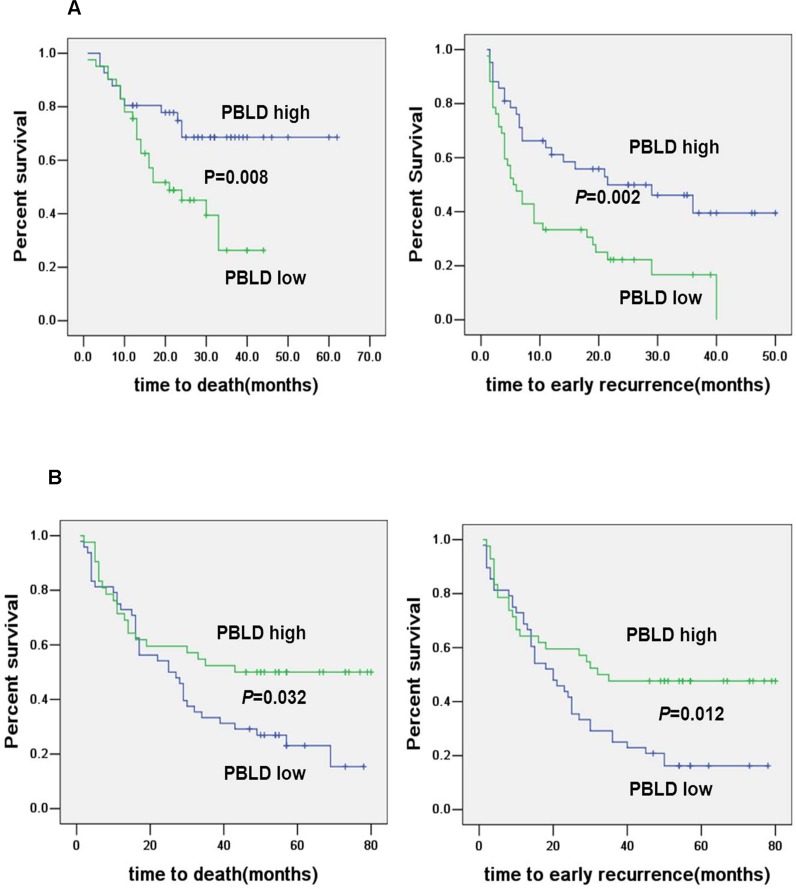
Kaplan–Meier curves for OS and RFS by PBLD expression Decreased PBLD expression predicted poor prognosis in HCC patients **A**. which was further validated by another independent cohort **B**.

Kaplan-Meier analysis revealed that decreased expression of PBLD was significantly associated with shorter OS and RFS as illustrated in Figure 2A. Patients with high PBLD expression had a 3-year OS of 68.6% compared with 26.3% for patients with low PBLD. Corresponding 3-year RFS rate was 61.1% versus 39.5% for high versus low PBLD. Our univariate analysis indicated that serum AFP level, vascular invasion, intrahepatic metastasis, tumor number, differentiation, AJCC stage and PBLD expression were significant prognostic factors for OS and/or RFS (Table 2). A low PBLD expression was significantly associated with both shorter RFS (HR 0.447, 95% CI 0.260-0.769, *P* = 0.004) and OS (HR 0.384, 95% CI 0.165-0.894, *P* = 0.027). Moreover, multivariate analysis suggested that PBLD was an independent prognostic factor for both RFS (HR 0.502, 95% CI 0.282-0.891, *P* = 0.019) and OS (HR 0.364, 95% CI 0.138-0.957, *P* = 0.040) (Table 2).

**Table 2:** Univariate and multivariate analyses of factors associated with RFS and OS in patients with HCC

	RFS		OS	
	HR (95%CI)	*P* value	HR (95%CI)	*P* value
**Univariate analysis**				
Age, yrs (<50 vs. ≥50)	0.994 (0.973-1.015)	0.553	0.659 (0.315-1.534)	0.368
Gender (male vs. female)	1.160 (0.046-2.910)	0.753	1.037 (0.307-3.507)	0.953
HBsAg (negative vs. positive)	1.996 (0.622-6.400)	0.245	1.627 (0.383-6.913)	0.509
HBeAg (negative vs. positive)	0.929 (0.420-2.054)	0.856	2.457 (0.831-7.262)	0.104
AFP,ng/ml (≤400 vs. >400)	1.847 (1.081-3.156)	**0.025**	3.016 (1.326-6.859)	**0.008**
Liver cirrhosis (absent vs. present)	0.886 (0.493-1.594)	0.686	0.792 (0.341-1.838)	0.587
Vascular invasion (absent vs. resent)	2.183 (1.167-4.084)	**0.015**	4.228 (1.798-9.942)	**0.001**
Intrahepatic metastasis (absent vs. present)	3.674 (2.056-6.566)	**0.000**	3.079 (1.270-7.764)	**0.013**
Tumor size, cm (≤5 vs. >5)	2.884 (1.358-6.125)	**0.006**	1.720 (0.644-4.589)	0.279
Tumor number (single vs. multiple)	3.464 (1.932-6.211)	**0.000**	2.622 (1.038-6.622)	**0.041**
Differentiation (poor/moderate vs. well)	1.986 (1.084-3.641)	**0.026**	3.729 (1.391-10.000)	**0.009**
AJCC stage(I-II vs. III-IV)	3.131 (1.805-5.430)	**0.000**	5.081 (2.273-11.360)	**0.000**
PBLD (low vs. high)	0.447 (0.260-0.769)	**0.004**	0.384 (0.165-0.894)	**0.027**
**Multivariate analysis**				
Tumor size, cm (≤5 vs. >5)	2.839 (1.270-6.345)	**0.011**		
AJCC stage (I-II vs. III-IV)	2.400 (1.137-5.066)	**0.022**	9.349 (1.754-49.842)	**0.009**
PBLD (low vs. high)	0.502 (0.282-0.891)	**0.019**	0.364 (0.138-0.957)	**0.040**

The prognostic role of tumor expression of PBLD was further validated in an independent cohort of 90 HCC patients. Kaplan-Meier analysis showed that PBLD was significantly associated with RFS (*P* = 0.012) and OS (*P* = 0.032) (Figure 2B). Multivariate analysis also indicated that PBLD was a powerful prognostic marker for recurrence (HR 0.490, 95% CI 0.285-0.841, *P* = 0.010) and survival (HR 0.528, 95% CI 0.303-0.922, *P* = 0.025) (Supplementary Table 2).

### Targeted overexpression of PBLD results in decreased growth, migration and invasion of HCC cells *in vitro*

**Figure 3:**
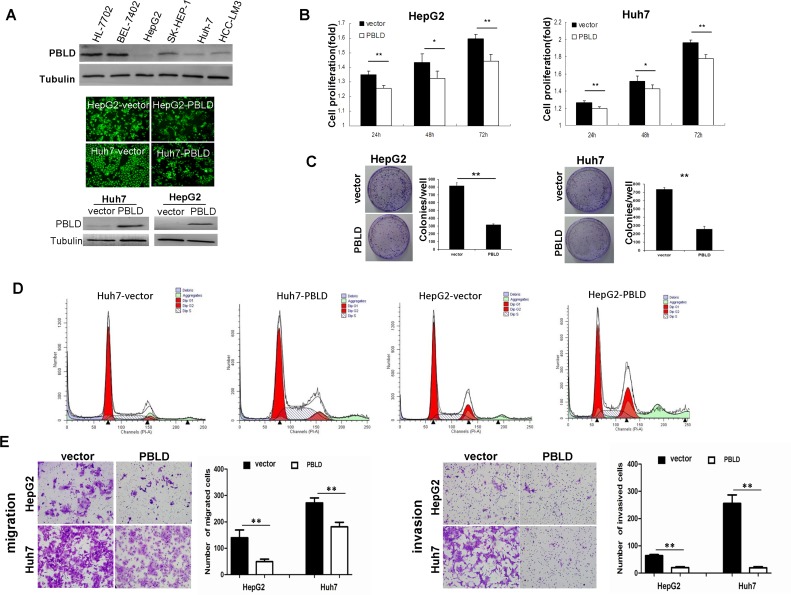
PBLD inhibited proliferation, migration and invasion *in vitro* **A**. Expression of PBLD was detected by western blotting in the human liver cell-line HL-7702 and HCC cell-lines BEL-7402, HepG2, SK-HEP-1, Huh-7 and HCC-LM3 (top). Representative fluorescence images of Huh7 and HepG2 cell-lines stably transfected with PBLD_pEGFP-N1 or empty vector (pEGFP-N1) (middle). PBLD protein expressions of Huh7 and HepG2 were efficiently upregulated after transfection as detected by western blotting (bottom). (**B**., **C**.) PBLD overexpression inhibited cell proliferation *in vitro* as analyzed by the cck-8 assay and colony formation assay, respectively. **D**. Upregulation of PBLD in Huh7 and HepG2 resulted in inhibition of cell cycle progression as detected by flow cytometry. **E**. PBLD also inhibited HCC cell migration (left) and invasion (right) *in vitro*. (*P < 0.05, **P < 0.001).

We chose HCC cell lines HepG2 and Huh7 for PBLD-targeted overexpression because of its low abundance of PBLD as indicated in Figure 3A (top panel). The amounts of PBLD protein were considerably upregulated in HCC cells after transfection with the PBLD gene, as determined by western blotting (Figure 3A). The mRNA expression levels of PBLD were upregulated 216-fold, 198-fold in HepG2_PBLD and Huh7_PBLD cells, respectively, compared with cells transfected with empty vector (pEGFP-N1) by qRT-PCR (Supplementary Figure 1).

We next determined the effects of PBLD overexpression on cell growth in HepG2 and Huh7 *in vitro*. The results of the CCK-8 proliferation assay showed that PBLD upregulation significantly inhibited the proliferation rate of HCC cells (*P* < 0.01; Figure 3B). Colony formation analysis consistently showed that PBLD significantly decreased the quantity and size of HepG2 and Huh7 cell colonies (Figure 3C). Cell cycle analysis revealed that PBLD overexpression caused a considerable inhibition of cell cycle progression, a characteristic decreased G1 phase, leading to a selective accumulation of cells in the S phase compared with control in Huh7 cells and G2/M phase arrest in HepG2 cells (Figure 3D) (Supplementary Table 3). We then tested the effects of PBLD silencing in relation to cell proliferation in the PBLD overexpressing cell lines HL-7702 and BEL-7402 *in vitro*. The results of the CCK-8 proliferation assay showed that PBLD downregulation significantly enhanced the proliferation rate of HCC cells (*P* < 0.05; Supplementary Figure 2). These results all suggested that PBLD inhibited the growth of HCC cells *in vitro*.

To investigate the effects of PBLD on the migration and invasion of HCC cells, we carried out transwell migration and invasion assays. As illustrated in Figure 3E, HepG2 and Huh7 cells migrated slower and had less ability to invade through the Matrigel-coated inserts when PBLD was upregulated. Taken together, the results mentioned above suggest that PBLD may be a potent tumor suppressor gene, and more investigation still need in *vivo*.

### Upregulated expression of PBLD retards tumor growth and angiogenesis *in vivo*

To explore whether the level of PBLD expression could affect hepatocarcinogenesis, PBLD_pEGFP-N1 or empty vector stably-transfected HepG2 cells were respectively inoculated into the left flank of nude mice. After 30 days, the xenograft tumors formed in HepG2_PBLD group were substantially smaller than those in the control group (Figure 5A). We also found that the expression of CD31 which indicated vascularization was significantly inhibited in tumor xenografts formed from cells transfected with PBLD_pEGFP-N1 using immunohistochemistry staining (Figure 5D). Immunofluorescence staining was then performed to evaluate the PBLD expression in selected xenograft tumor tissues. The results showed that the levels of PBLD expression in tumor tissues formed from PBLD_PEGFP-N1 transfected cells were higher than those from empty vector (pEGFP-N1) transfected cells (Figure 5B). These results *in vivo* experiments further indicate that overexpression of PBLD could inhibit HCC cell growth.

### PBLD inhibits multiple signaling pathways associated with tumor progression

**Figure 4:**
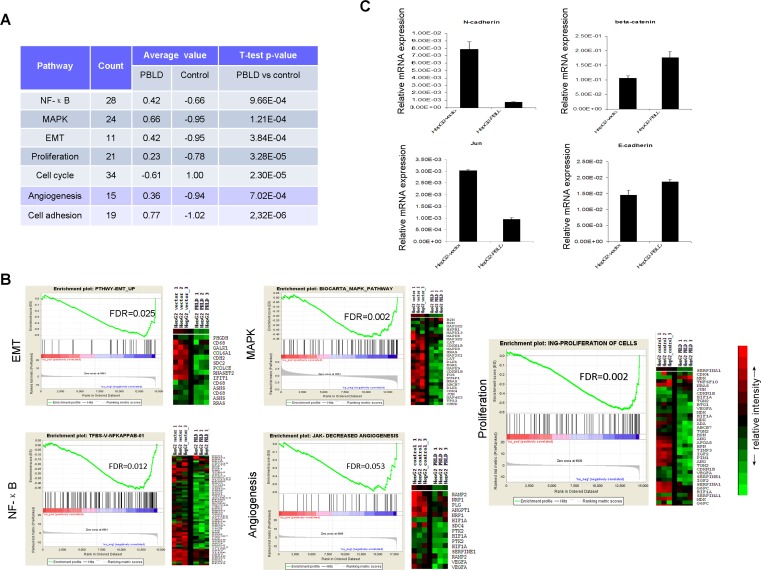
Whole genome microarray analysis of gene expression in HepG2 cells transfected with PBLD_pEGFP-N1 or empty vector **A**. Functional annotation was carried out using gene lists submitted to GSEA and DAVID. **B**. Expression of MAPK, NF-κB, EMT, angiogenesis and proliferation pathway genes were down-regulated by PBLD overexpression. GSEA results for all fold changes calculated between HepG2_PBLD and control cells were shown on right, and heatmap of significant genes in the each signaling pathway were displayed on left. Red represents high expression levels and green represents low expression levels. **C**. Gene expression of several marker genes (*E-cadherin, β-catenin, JUN, and N-cadherin*) was further confirmed by qRT-PCR.

Based on the observations summarized above, we performed Illumina's whole-genome expression arrays to identify the downstream genes mediating the effects of PBLD in HepG2 cells. Compared with the cells stably transfected with empty vector, specific deregulations of 289 known genes (96 upregulated genes; 193 downregulated genes) were identified in HepG2_PBLD cells. Concordant with the results presented in Figure 3, gene set enrichment analysis (GSEA) showed that these deregulated genes was associated with cell cycle, cell adhesion, proliferation, and several tumorigenesis-related signaling pathways. Pathway analysis of differentially expressed genes further highlighted the functional networks, including MAPK, NF-κB, EMT, angiogenesis and others. From the heat map, a clear stepwise decrease in average gene expression can be seen from HepG2_PBLD to control cells (Figure 4A & 4B). Gene expression of several marker genes (e.g., downregulation of *E-cadherin* and *β-catenin*, and *JUN*, and upregulation of *N-cadherin*) was confirmed by qRT-PCR (Figure 4C).

**Figure 5:**
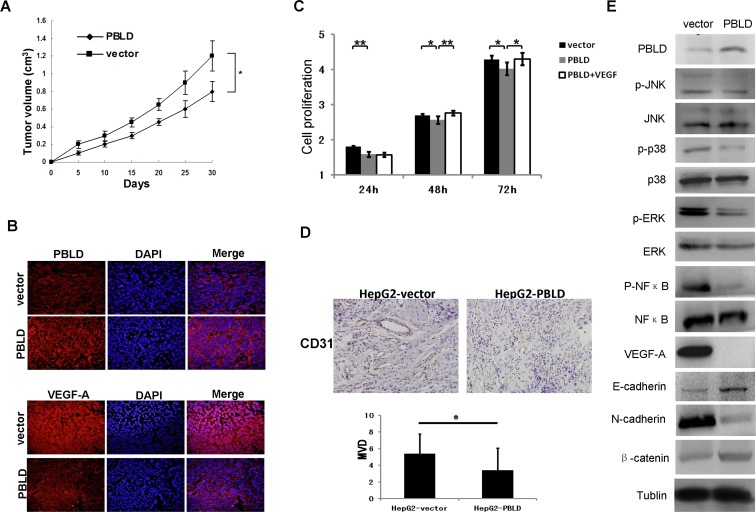
Upregulation of PBLD inhibited the growth of HepG2-derived xenografts *in vivo* via multiple signaling pathways **A**. Growth curves of the tumor xenografts in nude mice. **B**. Representative immunofluorescence staining of the PBLD or empty vector expressing HepG2 tumor xenografts stained by anti-PBLD antibody (upper panel) and anti-VEGF-A antibody (bottom panel). Upregulation of PBLD inhibited the expression of VEGF-A in tumor xenografts. Original magnification, ×400. **C**. PBLD overexpression inhibited cell proliferation *in vitro* as analyzed by the cck-8 assay, and VEGF-A overexpression can revert the effects of PBLD overexpression in vitro as analyzed by the cck-8 assay; **D**. Representative immunohistochemical staining of the PBLD or empty vector expressing HepG2 tumor xenografts stained by anti-CD31 antibody, the expression of CD31 which indicated vascularization was significantly inhibited in tumor xenografts formed from cells transfected with PBLD_pEGFP-N1. (*P*<0.05, original magnification, ×400) **E**. Representative western blot of tumor xenografts derived from HepG2_PBLD or the control cells. PBLD overexpression influenced the activation and expression of multiple genes related to p38, ERK, NF-κB and EMT, but not JNK in tumor xenografts as determined by western blot analysis. (*P < 0.05, **P < 0.001).

Analysis of microarray data prompted us to further confirm the effect of PBLD overexpression on the functional networks in HepG2-derived xenografts harboring PBLD_pEGFP-N1 or control empty vector. We found that the expression of VEGF-A was significantly inhibited in tumor xenografts formed from cells transfected with PBLD_pEGFP-N1 using immunofluorescence staining (Figure 5B) and western blotting analysis (Figure 5E) which was closely associated with tumor growth and angiogenesis. Further more, we also proved that the proliferation rate of HepG2_PBLD was enhanced after incubation with VEGF by CCK-8 assay *in vitro* (Figure 5C). These results indicated that the anti-proliferative effects of PBLD overexpression were mediated by VEGF-A. Meanwhile, although western blot did not suggested an effect on the total P38, extracellular signal-regulated protein kinases 1/2 (ERK1/2), and NF-κB protein, PBLD overexpression resulted in a significant reduction of active phosphorylated proteins in PBLD-xenograft tissues. In contrast, the phosphorylation level of JNK was incomparable between the PBLD-expressing and control xenografts, suggesting that PBLD selectively regulates the ERK1/2 or p38 MAPK pathway rather than JNK (Figure 5E). We further proved that impairment of ERK1/2 pathways by inhibitor U0126 resulted in similar effects (decreased growth, migration and invasion of HCC cells) such as those induced by PBLD overexpression *in vitro* (Supplementary Figure 4). We also investigated the differences of EMT markers in two group xenografts. Western blotting analysis revealed that HCC cells with forced PBLD expression in xenografts displayed upregulated E-cadherin, β-catenin, and downregulated N-cadherin (Figure 5E). These data suggested that multiple tumor progression–related signaling pathways including p38 MAPK, ERK, NF-κB, EMT and VEGF were coordinated by PBLD, and formed functional networks in HCC.

## DISCUSSION

PBLD was first isolated and sequenced from a human liver cDNA library by Iriyama *et al.* in 2001 [13]. Recently, accumulating genomic and proteomic data have suggested that decreased expression of PBLD is involved in the progression of HCC [7–10]. Using IHC and western blotting analysis, previous study has shown that expressions of PBLD were often lower in HCC than that in adjacent non-tumorigenic liver tissues [10]. The prognostic value and functional implications of PBLD in HCC, however, remain largely undefined. In current study, besides confirming the expression of PBLD was downregulated in HCC tissues, we further revealed that low PBLD expression was significantly correlated with poor tumor differentiation and an advanced stage, and decreased expression of PBLD predicted poor prognosis for patients with HCC after hepatectomy. Furthermore, multivariate analysis revealed that PBLD was a prognostic predictor for RFS and OS independent of other clinicopathologic variables. Consistent with our findings, Xu *et al.* found that in a screening study among 465 proteins by proteomics, only nine unique proteins for HCC including PBLD were identified to be downregulated in HCC tissues with PVTT compared with that without PVTT [11]. In addition, Das *et al.* found that HCCs from patients who developed HCC recurrence ≤3 years from liver transplantation showed lower expression level of PBLD than that from patients who did not have recurrent HCC [12]. Collectively, our findings and previous observations strongly suggested that PBLD may serve as a potent prognostic marker for patients with HCC.

To extend our clinical studies and investigate the function of PBLD in the progression of HCC, we constructed two stably transfected HCC cell lines overexpressing PBLD. Results revealed that PBLD attenuated the proliferation ability of HepG2 and Huh7 *in vitro* and suppressed the growth of xenografts in athymic nude mice. Meanwhile, PBLD silencing could enhance the proliferation rate in the PBLD overexpressing cell lines HL-7702 and BEL-7402 *in vitro*. The growth inhibition of tumor cells was often related to the induction of biosynthesis of DNA and RNA, cell cycle arrest, apoptosis, tumor angiogenesis and host immune function trigger by a series of genes [16]. In our study, the anti-proliferation effects of enhanced PBLD may be closely linked to the cell cycle arrest at the S or G2/M phase analyzed by flow cytometry. Furthermore, several proliferation-related genes such as *TGM2*, *VEGF-A*, *HIF1A*, *TIMP3*, as well as cell cycle-regulated genes such as *CDK4*, *CCNA2*, *CCND3*, *AURKA*, and *CDKN1B* were shown differential expression through microarray analysis (Figure 4B; Supplementary Table 4). These observations supported the conclusions from our clinical data that hepatic PBLD was involved in the growth of HCC cells and may play a critical role in liver tumorigenesis and progression as a tumor suppressor. Consistent with our results, a recent research on gastric cancer reported that overexpression of PBLD could suppress the growth and invasion of SGC7901 cells, while knockdown of this gene demonstrated the opposite effects, and tumorigenicity experiments showed that PBLD inhibited gastric cancer cell growth in *vivo* [15].

In the pathogenesis of HCC, multiple and diverse mechanisms have been reported to cause the aberrant proliferation and dedifferentiation of hepatocytes which lead to the subsequent development of malignant neoplasia [17]. Here we showed that inactivation of several tumorigenesis-related signaling pathways, including MAPK, NF-κB, EMT, angiogenesis and others, were identified following overexpression of PBLD by microarray analysis. All solid tumors are angiogenesis-dependent, that is, their growth beyond a certain size requires the formation of new blood vessels for the transport of nutrients and oxygen [18]. HCC is one of the most vascularized solid human tumors [19] and the extent of angiogenesis in HCC correlates tightly with the growth and metastasis of tumors [20, 21]. Although numerous growth factors are involved, vascular endothelial growth factor (VEGF), particularly VEGF-A, has been shown to play a pivotal role in tumor angiogenesis [22]. VEGF induces new vessel formation and tumor growth by inducing mitogenesis and chemotaxis of normal endothelial cells and increasing vascular permeability [23], which plays a crucial role in the proliferation, migration, adhesion and survival of human HCC cells [24–26]. Therefore the tumor-suppressive effects of PBLD overexpression are likely mediated through inhibition of angiogenesis, which is supported by several direct and indirect observations. First, the expression of CD31 which indicated vascularization was significantly inhibited in tumor xenografts formed from cells transfected with PBLD_pEGFP-N1. Second, we proved that VEGF-A overexpression can revert the effects of PBLD overexpression *in vitro*. Third, our microarray analysis indicated that overexpression of PBLD influenced multiple downstream genes related to angiogenesis, such as *VEGF-A*, *HIF1A*, *ANXA2*, *TGM2*, *ANG* and *CDH2*. Forth, our results show that the expression of VEGF-A was significantly inhibited in PBLD-overexpressing xenografts by western blot and immunofluorescence staining. Fifth, it has been demonstrated that knockdown of VEGF attenuated the migration, invasion, adhesion and survival of HCC cells [27]. Besides angiogenesis, we further found that PBLD was closely associated with key markers of EMT, such as E-cadherin, β-catenin, vimentin, and N-cadherin. Because EMT also plays an important role in HCC invasiveness and metastasis [28–30], a negative correlation between PBLD expression and EMT process may provide an explanation for the action of PBLD depletion in HCC malignancy. What's more, VEGF-A promotes an EMT by the decreased E-cadherin and increased expression of N-cadherin and vimentin [31].

Another novel finding in this study was that upregulated PBLD greatly attenuated NF-kB transcriptional activity, and MAPK phosphorylation in HCC cells. The progression from normal cells to cancer cells is, among others, influenced by environmental and extracellular factors. The extracellular factors involved in this process include multiple signalings, among which MAPK and NF-κB are found to be upregulated in HCC [32]. Activated MAPK/NF-κB plays a causative role in malignant transformation and progression [33]. Recent studies have suggested that NF-κB regulates the expression of multiple genes involved in tumor spread and metastasis, including VEGF, and blockade of NF-κB can downregulate VEGF and inhibit angiogenesis [34, 35]. In addition, VEGF-dependent angiogenesis requires p38MAPK activation, and VEGFR blocker (Sorafenib), which has shown benefit in patients with HCC, induces cells apoptosis through RAF/MEK/ERK and c-Jun NH2-terminal kinase pathways in human pancreatic cancer cells [26]. Consistent with our findings, a negative correlation between PBLD expression and p38MAPK has been documented by a recent study on the ulcerative colitis [36]. In this study we proved that impairment of ERK1/2 pathways by inhibitor U0126 resulted in similar effects such as those induced by PBLD overexpression *in vitro*. Therefore, given the regulatory role in the inhibition of tumorigenesis-related signaling pathways, we assumed that PBLD might represent a promising therapeutic target for cancer progression mainly via ERK1/2 pathways and VEGF-A.

In conclusion, we identified a frequent deletion of PBLD in human HCC tissues, and this deletion was an independent predictor of poor prognosis for patients with HCC. Elevated PBLD expression may reduce HCC growth and invasion through inactivation of several tumorigenesis-related signaling pathways, including VEGF-A, ERK-MAPK, NF-κB, EMT and angiogenesis, thereby providing a potential therapeutic target for HCC (Supplementary Figure 5).

## MATERIALS AND METHODS

### Patients and tissue samples

This study was reported in accordance with REMARK guidelines [37, 38]. The institutional ethics committee of Guangzhou First People's Hospital approved all protocols and all enrolled subjects gave their written informed consent. Fresh HCC tissue samples, together with matched adjacent non-tumorous tissues, were collected from a total of 108 patients, who underwent surgical resection at our institute between 2008 and 2011. Detailed clinicopathological characteristics of HCC patients are presented in Table 1. Tumor differentiation was based on the criteria proposed by Edmonson and Steiner [39]. Tumor stage was defined according to American Joint Committee on Cancer/International Union against Cancer tumor-node-metastasis classification system [40].

Patient follow-up was completed by December 31, 2013. Of the 108 patients, 24 (19%) were lost to follow-up. The median follow-up period was 36 months (range, 13–64 months). By the end of the follow-up period, 48 patients (56.1%) had died. Tumor recurrences were confirmed based on typical imaging appearances in computed tomography scans and/or MRI and an elevated AFP level.

### Western blotting and immunohistochemical assay

Liver specimens taken from a total of 16 patients and HCC cells were subjected to Western blotting and proteins extracted from the specimens were resolved in 12% SDS-PAGE and electro-transferred onto PVDF membranes (350 mA for 1 h). All blots were scanned using a GeneGnome HR Bioimaging system (Syngene, Bristol, UK). Immunohistochemical staining was conducted using standard procedure detailed in Supplementary Methods, and scoring was performed independently by three investigators as previously described [41]. Primary antibodies used in this study are listed in the Supplementary Methods section of this typescript.

### Quantitative real-time PCR (qRT-PCR)

Total RNA was extracted from tissues using Trizol (Invitrogen, Gaithersburg, MD, USA) according to the manufacturer's protocol. qRT-PCR was carried on cDNA obtained from total RNA using the cDNA Archive Kit according to the manufacturer's protocol (Applied Biosystems, Foster City, CA, USA). qRT-PCR reactions were performed in triplicate using the SYBR Green system on Roche Light Cycler 480 Real Time PCR System (Roche Diagnostics, Mannheim, Germany). Relative gene expression levels were calculated by using the 2^−ΔΔCt^ method. The ΔCt value of each sample was calculated using *GAPDH* as an endogenous control gene.

### Cell culture and transfection

The human liver immortal cell-line HL-7702, and the HCC cell-lines BEL-7402, HepG2, SK-HEP-1, Huh-7 and HCC-LM3 were obtained from the Shanghai Institute of Biochemistry and Cell Biology (Shanghai, China). These cell-lines were maintained in Dulbecco's modified Eagle's medium (DMEM; Invitrogen, USA) that was supplemented with 10% fetal calf serum (FCS; Hyclone, USA), 100 IU/ml penicillin and 100 μg/ml streptomycin, in 5% CO_2_ at 37°C. The cells were pretreated with the DMSO vehicle control, ERK1/2 inhibitor U0126 (10 μM, Selleck Chemicals, Houston, TX, USA), or Recombinant Human VEGF165 (50 ng/ml, R & D Systems, Minneapolis, MN) for 24h followed by the indicated experiments.

Genechem (Shanghai, China) constructed the plasmid PBLD_pEGFP-N1 and the expression plasmid pEGFP-N1 vector. Lipofectamine-2000 (Invitrogen, Carlsbad, CA, USA) was used to transfect PBLD_pEGFP-N1 or pEGFP-N1 vector into HepG2 and Huh7 cells following manufacturer's protocol. Approximately 48 hours after transfection, drug selection was carried out using 800 μg/ml G418 (Mpbio, USA) to obtain stable transfectants, which we named HepG2_PBLD, Huh7_PBLD, HepG2_vector, and Huh7_vector. Transfection of control siRNA and PBLD siRNA (GenePharma, Shanghai, China) was performed in HL-7702 and BEL-7402 cells using Lipofectamine 3000 transfection reagent (Invitrogen, Carlsbad, CA, USA) according to the manufacturer's instructions.

### Cell proliferation, cell cycle and colony formation assay

Cells seeded in 96-well plates were incubated with 100μl DMEM containing 10μl CCK-8 (Beyotime, China) at 37°C for 2 hours. Proliferation rates were determined at 24, 48, and 72 hours after having been seeded into culture plates. Each time-point was done in replicates of six wells. Cell cycle distribution was analyzed by flow cytometry. HepG2 and Huh7 cells stably transfected with PBLD or empty vector were trypsinized, fixed in 70% ethanol, and incubated with 0.5 mg/ml of propidium iodide (PI) along with 0.1 mg/ml of RNase A (Calbiochem, San Diego, CA, USA). Data acquisition and analysis was achieved using a BD LSRFortessa (BD Biosciences, San Jose, CA, USA) with ModFit software (Verity Software House, USA). For colony formation assay, HepG2 and Huh7 cells stably transfected with PBLD or empty vector were plated in 6-well plates at a density of 1,000 cells per well. After 14 days, cells were washed with PBS, fixed in 4% paraformaldehyde for 20 minutes, and stained in Giemsa for 20 minutes. Colonies that consisted of >50 cells were scored. Each experiment was repeated at least three times.

### Cell migration and invasion assay

After starvation for 12 hours, cells were harvested for migration and invasion assay, which was carried out in 24-well plates with an 8-μm pore size transwell microporous membrane (3422, Corning, USA). The membrane for the invasion assay was covered with 40 ul of the BD Matrigel (diluted 1:8 with serum-free medium) in advance. Then, 200 μl cell suspension (containing 1×10^5^ cells for migration assay and 6×10^5^ cells for invasion assay) with serum-free medium was seeded in the upper chamber while the lower chamber was covered with 500 ul medium supplemented with 20% fetal calf serum. After 48 hours of incubation at 37°C in an atmosphere of 5% CO_2_ in air, the cells were washed with PBS and fixed in 4% paraformaldehyde for 10 minutes. Then cells in the upper surface of the membrane were removed with a cotton swab and the migrated cells on the bottom surface were stained with 0.5% crystal violet at 37°C for 30 minutes. The membrane was removed and mounted onto glass slides. Cells in five random fields were counted with a light microscope and the mean value was recorded.

### Microarray analysis

Whole-genome gene expression (Illumina, USA) was performed as per manufacturer's instructions. Data was collected using the Illumina Genome Studio software. Raw and normalized data can be accessed from the Gene Expression Omnibus (GEO) database (GSE53306). Functional annotation was carried out using gene lists submitted to a variety of on-line software tools including the DAVID gene functional classification tool and to GSEA (Gene Set Enrichment Analysis). Details of data analysis are described in greater detail in the Supplementary Methods section of this typescript.

### Tumorigenicity in nude mouse

4- to 6-week-old BALB/c male nude mice were purchased from Experimental Animal Center of Southern Medical University (Guangzhou, China). To assess the xenograft tumor growth, the mice were randomly assigned to two groups (5 for each group) and 4×10^6^ HepG2_PBLD or HepG2_vector cells were injected in the left flank of each mouse. Tumor volumes were examined every 5 days and calculated using the formula: length×width^2^/2. Mice were sacrificed by cervical dislocation after 30 days, and the tumors were frozen or fixed for immunofluorescence staining and western blot.

### Independent validation

A total of 90 HCC samples on the TMA (tissue microarray) with survival data obtained from China National Engineering Center for Biochip (Shanghai, China) were used as an independent validation cohort. The validation cohort had a follow-up time from 1 to 80 months (median, 29 months) after surgery. Immunohistochemistry was conducted to stratify patients into high PBLD group and low PBLD group. Clinicopathological characteristics are described in Supplementary Table 1.

### Statistical analysis

All statistical analyses were performed using SPSS.13.0 software. Difference between two independent groups was analyzed by Student's t test and Wilcoxon rank sum test. The correlation between PBLD expression and clinicopathologic characteristics was calculated using the Pearson chi-square tests. Recurrence-free survival (RFS) and overall survival (OS) rates were calculated using the Kaplan–Meier method and the significance was assessed using the log-rank test. Independent prognostic factors for OS and RFS were identified by the Cox proportional hazard regression model. Data were presented as mean ± SEM, and *P* value <0.05 was considered to be statistically significant.

## SUPPLEMENTARY MATERIAL FIGURES, METHODS AND TABLES


