# Supplementary material for: Decreased expression of PBLD correlates with poor prognosis and functions as a tumor suppressor in human hepatocellular carcinoma
Source: Oncotarget. 2015 Nov 22;7(1):524–37. doi: 10.18632/oncotarget.6358 (PMC4808015; doi:10.18632/oncotarget.6358)
Supplement: Supplementary file 1 [file oncotarget-07-0524-s001.pdf]

Decreased expression of PBLD correlates with poor prognosis and functions as a tumor suppressor in human hepatocellular carcinoma

Supplementary Material

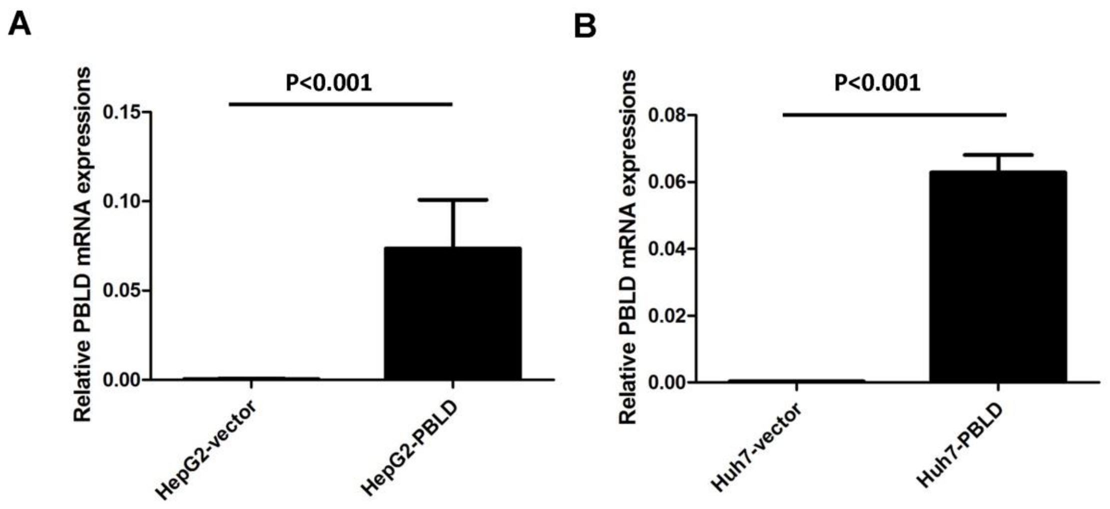

Supplementary Fig. 1. PBLD mRNA expression was significantly up-regulated in HepG2 and Huh7 cell lines after transfection detected by qRT-PCR. The expression level of PBLD was up-regulated 216-fold, 198-fold ($p < 0.01$) in HepG2_PBLD (A) and Huh7_PBLD (B) cell lines, respectively, compared with control pEGFP-N1 vector transfected cells.

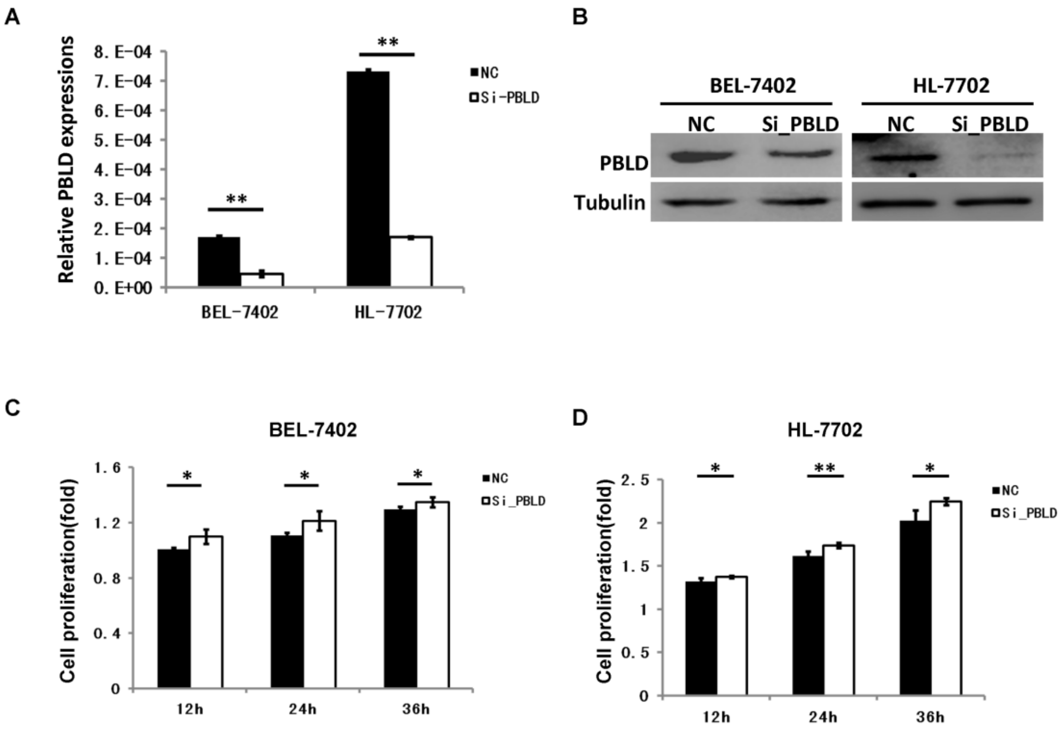

Supplementary Fig. 2. PBLD downregulation significantly enhanced the proliferation rate of HCC cells in vitro. (A) BEL-7402 and HL-7702 cell-lines transfected with PBLD siRNA or empty vector, PBLD mRNA expressions of BEL-7402 and HL-7702 cell-lines were efficiently downregulated after transfection as detected by qRT-PCR ($P<0.001$). (B) PBLD protein expressions of BEL-7402 and HL-7702 cell-lines were efficiently downregulated after transfection as detected by western blotting. (C, D) PBLD downregulation enhanced cell proliferation *in vitro* as analyzed by the cck-8 assay in BEL-7402 and HL-7702 cell-lines respectively. (*$P<0.05$, **$P<0.01$)

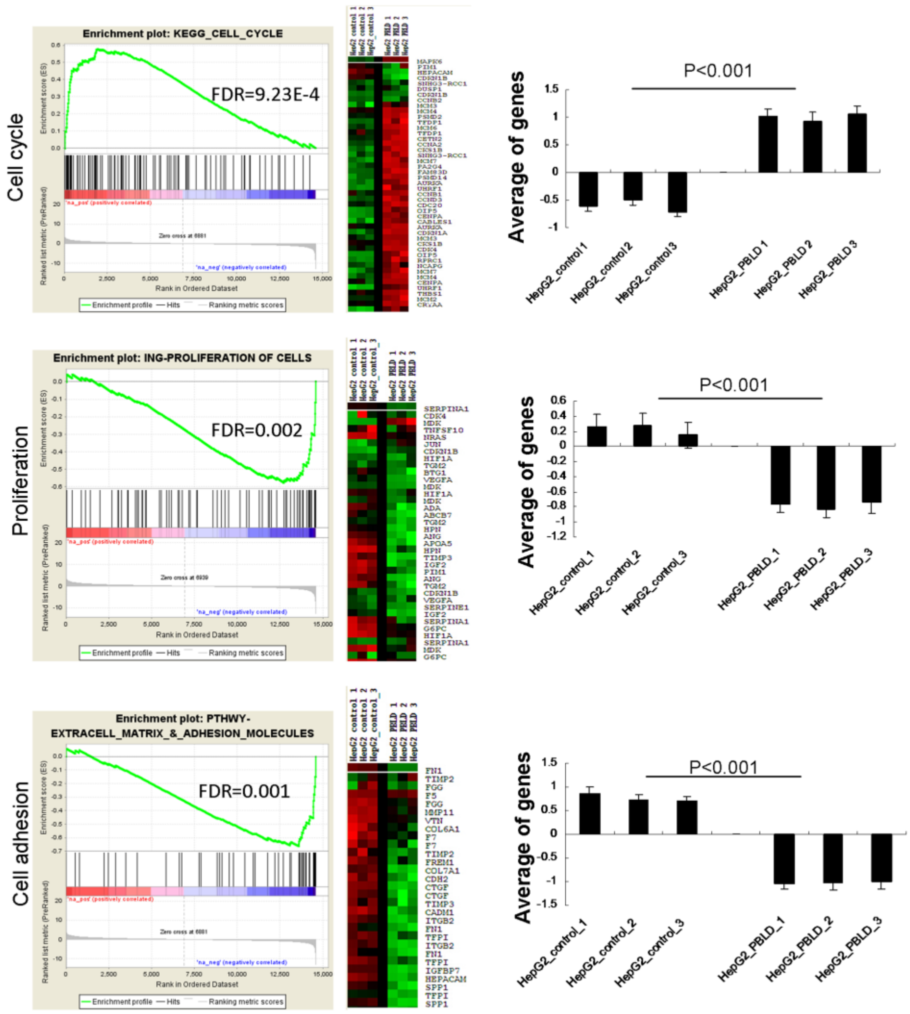

Supplementary Fig. 3.Cell cycle, proliferation and cell adhesion pathway related genes showed a marked deregulation in PBLD_HepG2 cells compared with cont rol. GSEA results for all fold changes calculated, the cell cycle (A), proliferation (B) and cell adhesion (C) pathway were all significantly deregulated; Heatmap displayed significant gene expression; the average gene expression for each sample in group were calculated (P<0.001).

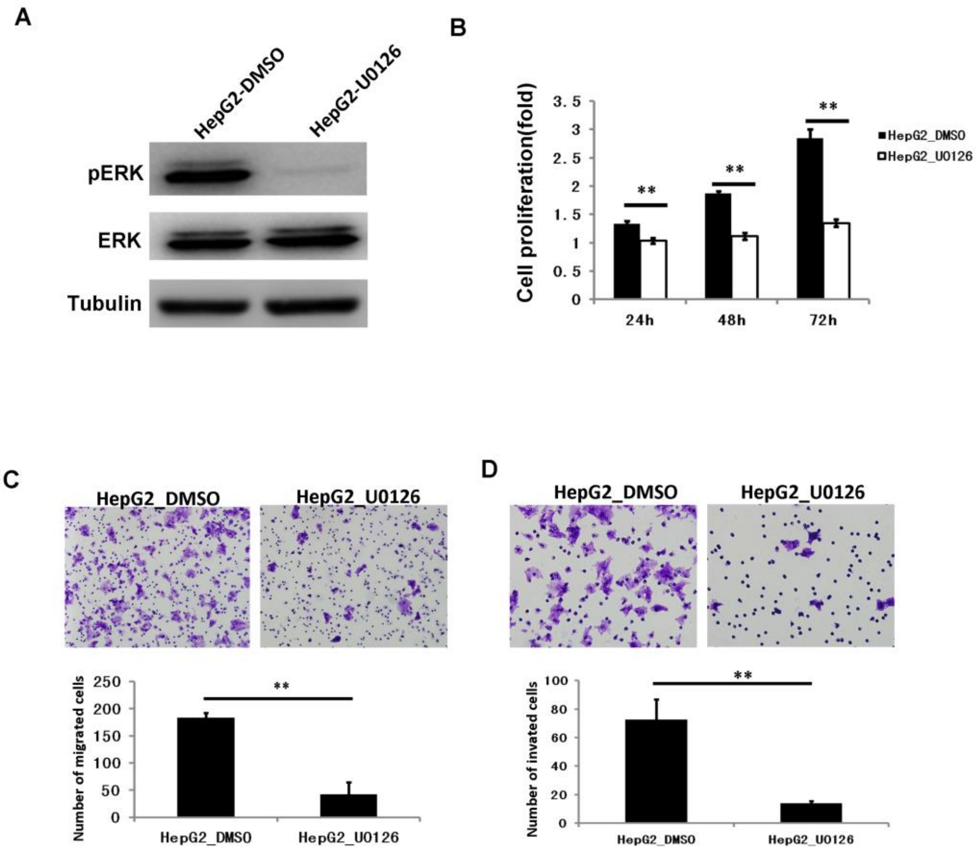

Supplementary Fig. 4. Impairment of ERK1/2 pathways inhibited proliferation, migration and invasion of HepG2 cell lines *in vitro* as similar as those induced by PBLD overexpression. (A) phospho-ERK protein expressions of HepG2 were efficiently downregulated after treated with U0126 as detected by western blotting. (B) Impairment of ERK1/2 pathways inhibited cell proliferation *in vitro* as analyzed by the cck-8 assay (P<0.001). (C, D) impairment of ERK1/2 pathways in HepG2 resulted in inhibition of cell migration and invasion *in vitro* (P<0.001).

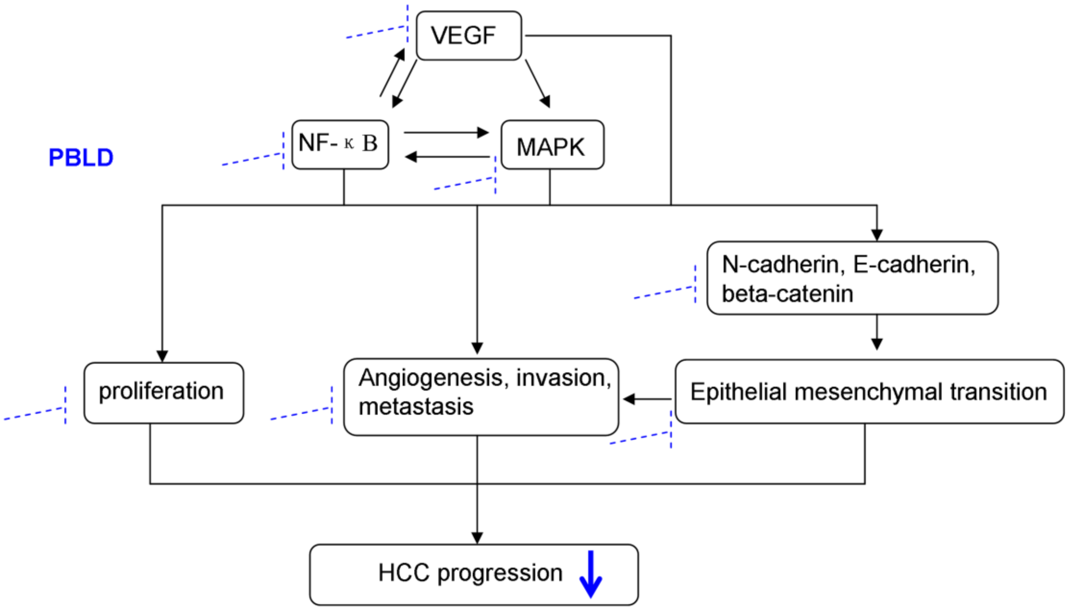

Supplementary Fig. 5.Several molecular mechanisms of PBLD have been recognized which involved in the progression of HCC. Elevated PBLD expression may reduce HCC growth and metastasis through inactivation of several tumorigenesis-related signaling pathways, including angiogenesis/VEGF, EMT, NF-κB, MAPK.

Supplementary Methods

Immunohistochemical assay

IHC staining for PBLD expression was done on 4-μm sections from paraffin-embedded tissue specimens. The sections were deparaffinized in xylene, and rehydrated by a series of graded ethanol rinse. Masked epitope retrieval was done by heating the sections in a microwave oven in 0.01 M sodium citrate buffer (pH 6.0) for 20 min to retrieve antigens. Endogenous peroxidase activity was terminated by incubation in 3% H_2O_2 for 20 min at room temperature. The sections were then incubated at 4ºC overnight with PBLD monoclonal mouse anti-human IgG (Santa Cruz Biotechnology, Santa Cruz, CA, USA) or CD31 polyclonal rabbit anti-human IgG (1:50; Abcam, Cambridge, UK) in a 1:50 dilution with 5% skimmed milk PBS buffer. Incubations with corresponding secondary antibodies were performed after the sections were allowed to stay at room temperature for 45 min. The antibody-antigen complexes were visualized with diaminobenzidine (DAB) alone and counterstained with haematoxylin. Finally, the sections were dehydrated in ethanol, cleared in xylene, and examined under light microscopy. Sections known to show positive staining for PBLD were included in each run, receiving either the primary antibody or PBS, as positive or negative controls. In all staining procedures, the positive controls showed clear staining, whereas there was no staining in the negative controls. For indirect immunofluorescence assay, the primary antibodies anti-PBLD monoclonal antibody (Santa Cruz Biotechnology, Santa Cruz, CA, USA) and anti-VEGF monoclonal antibody (Bioworld technology, co, Ltd, Nanjing, China) and secondary

antibody Cy3-goat anti-rabbit-IgG (EarthOx Life Science, Millbrae, CA, USA) were used.

The IHC slides were analyzed by three independent investigators without knowledge of clinicopathologic or biological information, one of whom was experienced pathologist (S. Liu). Every sample was given a score according to the intensity of the staining (no staining = 0, weak staining = 1, moderate staining = 2, strong staining = 3) and the extent of stained cells (<5% = 0, 5%–25% = 1, 26%–50% = 2, 51%–75% = 3, 76%–100% = 4). The percentage of cells at each intensity was multiplied by the corresponding intensity value to obtain an immunostaining score ranging from 0 to 12. The scores were combined to obtain an overall mean score. Using this assessment system, optimal cutoff values were identified by the mean score as follows: 0–3 (low), 4–8–12 (high). For quantification of angiogenesis, CD31-positive areas were counted at high power magnification in 15 random fields. Data was collected by two independent observers and the number of CD31-positive vessels was taken as the microvessel density (MVD) of individual groups.

Western blotting

Protein was extracted in RIPA buffer (Millipore, Billerica, MA, USA) containing 1% Halt Protease and Phosphatase Inhibitor Cocktails (Thermo Scientific, Rockford, IL, USA). The concentration of protein was determined using Pierce BCA Protein Assay Kit (Thermo Scientific, Rockford, IL, USA). Samples with equal amounts of total protein were separated by 12% SDS-PAGE and electrotransferred from the gel to

PVDF membranes (Bio-Rad Laboratories, Hercules, CA, USA). After blocking with 5% BSA (Sigma-Aldrich, St. Louis, MO, USA) in Tris-buffered saline (TBS) with 0.05% Tween-20 (Sigma-Aldrich, St. Louis, MO, USA) for 1 hour at room temperature with shaking, the membranes were probed subsequently by the primary antibodies mouse-anti-PBLD (1:1000; Abcam, Cambridge, UK), phospho-ERK (1:1000; Cell Signaling Technology, Inc, Beverly, MA; USA), total ERK (1:1000; Cell Signaling Technology, Inc, Beverly, MA; USA), phospho-p38 (1:1000; Cell Signaling Technology, Inc, Beverly, MA; USA), total p-38 (1:1000; Cell Signaling Technology, Inc, Beverly, MA; USA), phospho-JNK (1:1000; Cell Signaling Technology, Inc, Beverly, MA; USA), total JNK (1:1000; Cell Signaling Technology, Inc, Beverly, MA; USA), phospho-NF-κB (1:1000; Cell Signaling Technology, Inc, Beverly, MA; USA), total NF-κB (1:1000; Cell Signaling Technology, Inc, Beverly, MA; USA), VEGF-A (1:500; Bioworld technology, co, Ltd, Nanjing, China), E-cadherin (1:750; Bioworld technology, co, Ltd, Nanjing, China), N-cadherin (1:1000; Bioworld technology, co, Ltd, Nanjing, China), β-catenin (1:500; Bioworld technology, co, Ltd, Nanjing, China) at 4°C overnight. Tubulin (Tianjin Sungene Biotech Co., Ltd, Tianjin, China) or GAPDH (Tianjin Sungene Biotech Co., Ltd, Tianjin, China) was used as an internal control.

Quantitative real-time PCR

Total RNA was extracted from tissues using TRIZOL (Invitrogen Life Technologies Inc., Gaithersburg, USA) according to the manufacturer's protocol. One microgram of total RNA was reverse transcribed using a standard oligo

(deoxythymidine) primer in a total volume of 20 μl. Thirty nanogram of cDNA from each sample was used as template for PCR amplification with specific oligonucleotide primers in the Roche LightCycler 480 Real Time PCR System (Roche Diagnostics, Mannheim, Germany). PCR reactions were performed according to the manufacturer's instructions, using the SYBR Green qPCR Master Mix (DBI Bioscience, Shanghai, China). The following conditions were used for PCR: 95 °C for 15 s, 60°C for 20 s and repeated for 40 cycles. The identities of the PCR products were confirmed by melting temperatures and dissociation curves. For quantitation of gene expression, the fluorescence of the SYBR Green dye bound to the PCR products was measured after each cycle, and the cycle numbers were recorded when the accumulated signals crossed an arbitrary threshold (CT value). All PCR reactions were performed in duplicate when cDNA samples were available. To normalize this value, a ΔCT value was determined as the difference between the CT value for each gene and the CT value for GAPDH. For each gene, a ΔΔCT value was determined as the difference between the ΔCT value for each HCC tissue sample and the average ΔCT value for this gene obtained from the normal tissue samples. These ΔΔCT values were then used to calculate $2^{-\Delta\Delta CT}$ values as relative gene expression. The primer sequences for real-time PCR were as follows: PBLD sense, 5'- GGGTCTGCACACG CTGTTC-3' and antisense, 5'- TAATGTCAACCCTTCCGTCT -3'; JUN sense, 5'- CCAAAGGATAGTGCGATGTTT -3' and antisense, 5'- CTGTCCCTCTCCA CTGCAAC -3', E-cadherin sense, 5'- GAGTGCCAACTGGACCATTCAGTA -3' and antisense, 5'- AGTCACCCACCTCTAAGGCCATC -3', N-cadherin sense, 5'-

CGAATGGATGAAAGACCCATCC-3' and antisense, 5'- GGAGCCACTGCCTTC ATAGTCAA-3', beta catenin sense, 5'- GCTTGTTCGTGCACATCAGGATA -3' and antisense, 5'- GGCTCCGGTACAACCTTCAACTA-3, GAPDH sense, 5'- GAAGGT GAAGGTCGGAGT-3' and antisense, 5'-GAAGATGGTGATGGGATTTC-3'.

Microarray analysis

Total RNA was extracted using the Trizol Reagent method. Additional purification was performed on RNeasy columns (Qiagen, Valencia, CA 913555, cat. no. 74104). The quality of total RNA samples as assessed using an Agilent 2100 Bioanalyzer (Agilent Technologies, Palo Alto, CA).

RNA samples were labeled according to the chip manufacturer's recommended protocols. In brief, for Illumina, 0.5 µg of total RNA from each sample was labeled by using the IlluminaTotalPrep RNA Amplification Kit (Ambion, Austin, TX 78744-1832, cat. no. IL1791) in a two step process of cDNA synthesis followed by *in vitro* RNA transcription. Single stranded RNA (cRNA) was generated and labeled by incorporating biotin-16-UTP. 0.75 µg of biotin-labeled cRNA was hybridized (16 hours) to Illumina HumanHT-12_v4 BeadChips (Illumina, San Diego, CA, USA). The hybridized biotinylated cRNA was detected with streptavidin-Cy3 and quantified using Illumina'sBeadStation 500GX Genetic Analysis Systems scanner.

Preliminary data analysis of the scanned data was performed using IlluminaBeadStudio software which returned single intensity data values/gene following the computation of a trimmed mean average for each probe type

represented by a variable number of bead probes/gene on the array. Z-transformation for normalization was performed on each Illumina sample/array on a stand-alone basis, and significant changes in gene expression between class pairs were calculated by Z test. Significant gene lists were calculated by selecting genes which satisfied a significance threshold criteria of Z test p-values less than or equal to 0.001 (10^{-3}), a false discovery rate less than or equal to 0.1, and a fold change ± 1.5 or greater.

Supplementary Table1. The clinicopathological characteristics of 90 patients with HCC in the validated cohort.

**Variables**	**No. of patients(n=90)**
**Median age (y; range)**	54(25-73)
**Gender**	
Male	78
Female	12
**Liver Cirrhosis**	
Absent	56
Present	34
**Vascular invasion**	
Absent	82
Present	8
**Intrahepatic metastasis**	
Absent	78
Present	11
**Tumor size**	
≤5 cm	38
＞5 cm	51
**Tumor number**	
Single	78
Multiple	11
**Tumor differentiation**	
Well- Moderate	56
Poor	34
**TNM stage**	
I/II	45
III/IV	45

Table Legend: A total of 90 HCC samples on the TMA with clinicopathological characteristics listed above were used as an independent validation cohort.

Supplementary Table2. Univariate and multivariate analyses in the validated patients cohort.

	RFS		OS	
	**HR (95%CI)**	***P* value**	**HR (95%CI)**	***P* value**
**Univariate analysis**				
Age, yrs (<50 vs. ≥50)	0.987 (0.963-1.011)	0.291	0.993 (0.967-1.018)	0.568
Gender (male vs. female)	1.041 (0.495-2.188)	0.916	1.135 (0.537-2.397)	0.740
Liver cirrhosis (absent vs. present)	0.965 (0.576-1.618)	0.892	0.917 (0.535-1.570)	0.751
Vascular invasion (absent vs. resent)	2.547 (1.146-5.661)	**0.022**	3.002 (1.341-6.724)	**0.008**
Intrahepatic metastasis (absent vs. present)	1.659 (0.814-3.381)	0.163	1.734 (0.848-3.548)	0.132
Tumor size, cm (≤5 vs. >5)	2.089 (1.225-3.562)	**0.007**	2.157 (1.245-3.801)	**0.006**
Tumor number (single vs. multiple)	1.659 (0.814-3.381)	0.163	1.734 (0.848-3.548)	0.132
Differentiation (poor/moderate vs. well)	1.281 (0.770-2.129)	0.341	1.305 (0.773-2.202)	0.319
AJCC stage(I-II vs. III-IV)	2.505 (1.492-4.205)	**0.001**	2.552 (1.486-4.385)	**0.001**
PBLD (low vs. high)	0.522 (0.309-0.882)	**0.015**	0.563 (0.329-0.965)	**0.037**
**Multivariate analysis**				
AJCC stage (I-II vs. III-IV)	2.052 (1.065-3.953)	**0.032**	1.982 (1.006-3.905)	**0.048**
PBLD (low vs. high)	0.490 (0.285-0.841)	**0.010**	0.528 (0.303-0.922)	**0.025**

Table Legend: Univariate and multivariate analysis showed that PBLD was a prognostic predictor for both RFS and OS in another independent patients cohort.

Supplementary Table 3. Effects of PBLDoverexpression on cell cycle distribution in HepG2 and Huh7 cell lines by flow cytometry.

Groups	G0-G1 (%)	S phase (%)	G2/M (%)
Huh7-vector	67.28±0.76	23.36±2.36	9.31±3.05
Huh7-PBLD	50.73±1.86**	39.52±3.13**	9.93±3.30
HepG2-vector	59.98±2.76	22.42±2.21	17.60±0.62
HepG2-PBLD	46.53±0.99**	21.98±0.42	31.49±0.61**

NOTE: Values were presented as mean ± SD of three independent experiments. **P<0.01, compared to the corresponding vector-expressing transfectants.

Table Legend: Cell cycle analysis revealed that PBLD overexpression caused a considerable inhibition of cell cycle progression, a characteristic decreased G1 phase, leading to a selective accumulation of cells in the S phase compared with control in Huh7 cells and G2/M phase arrest in HepG2 cells.

Supplementary Table 4. Genes showing significantly different expression level regulated by PBLD overexpression in HepG2 cells.

Function	Gene symbol	Gene name	Expression change in HCC tumor*	GenBank No.	UniGene ID
NF-κB	**LGALS1**	Lectin, galactoside-binding, soluble, 1	↓	NM_002305.3	Hs.445351
	**TGM2**	Transglutaminase 2 (C polypeptide, protein-glutamine-gamma-glutamyltransferase)	↓	NM_004613.2	Hs.517033
	**TNFSF10**	Tumor necrosis factor (ligand) superfamily, member 10	↓	NM_003810.3	Hs.478275
	**BNIP3**	BCL2/adenovirus E1B 19kDa interacting protein 3	↓	NM_004052.2	Hs.144873
	**MAPK9**	Mitogen-activated protein kinase 9	↓	NM_002752.4	Hs.484371
	**FOS**	FBJ murine osteosarcoma viral oncogene homolog	↓	NM_005252.3	Hs.25647
	**EGR1**	Early growth response 1	↓	NM_001964.2	Hs.326035
	**JUN**	Jun B proto-oncogene	↓	NM_002229.2	Hs.25292
	**UBD**	Ubiquitin D	↓	NM_006398.3	Hs.44532
	**CTGF**	Connective tissue growth factor	↓	NM_001901.2	Hs.410037
	**EBI3**	Epstein-Barr virus induced 3	↓	NM_005755.2	Hs.501452
	**HSD3B7**	Hydroxy-delta-5-steroid dehydrogenase, 3 beta- and steroid delta-isomerase 7	↓	NM_025193.3	Hs.460618
	**MSX1**	Msh homeobox 1	↓	NM_002448.3	Hs.424414
	**UBE4B**	Ubiquitination factor E4B	↓	NM_001105562.2	Hs.593974
	**CHD6**	Chromodomain helicase DNA binding protein 6	↓	NM_032221.3	Hs.740645
	**DDR1**	Discoidin domain receptor tyrosine kinase 1	↓	NM_001954.4	Hs.631988
	**CD83**	CD83 molecule	↓	NM_004233.3	Hs.595133
	**MAML2**	Mastermind-like 2 (Drosophila)	↓	NM_032427.1	Hs.745167
	**UBE2H**	Ubiquitin-conjugating enzyme E2H	↓	NM_003344.3	Hs.643548
	**SDC4**	Syndecan 4	↓	NM_002999.3	Hs.632267
	**TCEA2**	Transcription elongation factor A (SII), 2	↓	NM_003195.4	Hs.505004
	**CPD**	Carboxypeptidase D	↓	NM_001304.4	Hs.446079
	**RIN2**	Ras and Rab interactor 2	↓	NM_001242581.1	Hs.472270

	**CXCL16**	Chemokine (C-X-C motif) ligand 16	↓	NM_022059.2	Hs.745037
	**SLC11A2**	Solute carrier family 11 (proton-coupled divalent metal ion transporters), member 2	↓	NM_001174125.1	Hs.505545
	**BMF**	Bcl2 modifying factor	↓	NM_001003940.1	Hs.591104
	**LTB**	Lymphotoxin beta (TNF superfamily, member 3)	↓	NM_002341.1	Hs.376208
	**RBMS1**	RNA binding motif, single stranded interacting protein 1	↓	NM_016836.3	Hs.470412
MAPK	**DUSP1**	Dual specificity phosphatase 1	↓	NM_004417.3	Hs.171695
	**JUN**	Jun B proto-oncogene	↓	NM_002229.2	Hs.25292
	**EGR1**	Early growth response 1	↓	NM_001964.2	Hs.326035
	**FOS**	FBJ murine osteosarcoma viral oncogene homolog	↓	NM_005252.3	Hs.25647
	**MAP2K1**	Mitogen-activated protein kinase kinase 1	↓	NM_002755.3	Hs.145442
	**DLK1**	Delta-like 1 homolog (Drosophila)	↓	NM_003836.5	Hs.533717
	**CDK4**	Cyclin-dependent kinase 4	↓	NM_000075.3	Hs.95577
	**ABCB7**	ATP-binding cassette, sub-family B (MDR/TAP), member 7	↓	NM_004299.4	Hs.370480
	**CDKN1B**	Cyclin-dependent kinase inhibitor 1B (p27, Kip1)	↓	NM_004064.3	Hs.238990
	**NRAS**	Neuroblastoma RAS viral (v-ras) oncogene homolog	↓	NM_002524.4	Hs.486502
	**MAP4K3**	Mitogen-activated protein kinase kinase kinase kinase 3	↓	NM_003618.3	Hs.655750
	**MAPK6**	Mitogen-activated protein kinase 6	↓	NM_002748.3	Hs.411847
	**TIMP3**	TIMP metallopeptidase inhibitor 3	↓	NM_000362.4	Hs.644633
	**MAPK9**	Mitogen-activated protein kinase 9	↓	NM_002752.4	Hs.484371
	**TP53**	Tumor protein p53	↓	NM_000546.5	Hs.437460
	**CAT**	Catalase	↓	NM_001752.3	Hs.502302
	**CHUK**	Conserved helix-loop-helix ubiquitous kinase	↓	NM_001278.3	Hs.198998
	**HRAS**	V-Ha-ras Harvey rat sarcoma viral oncogene homolog	↓	NM_005343.2	Hs.37003
	**MAP2K2**	Mitogen-activated protein kinase kinase 2	↓	NM_030662.3	Hs.465627
	**MEF2D**	Myocyte enhancer factor 2D	↓	NM_005920.3	Hs.314327
	**ELK1**	ELK1, member of ETS oncogene family	↓	NM_001114123.2	Hs.181128
	**MAPK3**	Mitogen-activated protein kinase 3	↓	NM_002746.2	Hs.861
	**MKNK2**	MAP kinase interacting serine/threonine kinase 2	↓	NM_017572.3	Hs.515032
	**MAP2K2**	Mitogen-activated protein kinase kinase 2	↓	NM_030662.3	Hs.465627

EMT	**PCOLCE**	Procollagen C-endopeptidase enhancer	↓	NM_002593.3	Hs.202097
	**RNASET2**	Ribonuclease T2	↓	NM_003730.4	Hs.529989
	**CDH2**	Cadherin 2, type 1, N-cadherin (neuronal)	↓	NM_001792.3	Hs.464829
	**CD68**	CD68 molecule	↓	NM_001251.2	Hs.647419
	**RRAS**	Related RAS viral (r-ras) oncogene homolog	↓	NM_006270.3	Hs.515536
	**COL6A1**	Collagen, type VI, alpha 1	↓	NM_001848.2	Hs.474053
	**PHGDH**	Phosphoglycerate dehydrogenase	↓	NM_006623.3	Hs.487296
	**GALK1**	Galactokinase 1	↓	NM_000154.1	Hs.407966
	**SDC2**	Syndecan 2	↓	NM_002998.3	Hs.1501
	**ASNS**	Asparagine synthetase (glutamine-hydrolyzing)	↓	NM_133436.3	Hs.489207
	**IFIT1**	Interferon-induced protein with tetratricopeptide repeats 1	↓	NM_001548.4	Hs.20315
cell cycle	**CKS1B**	CDC28 protein kinase regulatory subunit 1B	↑	NM_001826.2	Hs.374378
	**CABLES1**	Cdk5 and Abl enzyme substrate 1	↑	NM_138375.2	Hs.11108
	**OIP5**	Opa interacting protein 5	↑	NM_007280.1	Hs.661645
	**AURKA**	Aurora kinase A	↑	NM_198433.1	Hs.250822
	**CDC20**	Cell division cycle 20	↑	NM_001255.2	Hs.524947
	**CETN2**	Centrin, EF-hand protein, 2	↑	NM_004344.1	Hs.82794
	**CENPA**	Centromere protein A	↑	NM_001809.3	Hs.1594
	**CRYAA**	Crystallin, alpha A	↑	NM_000394.2	Hs.184085
	**CCNA2**	Cyclin A2	↑	NM_001237.3	Hs.58974
	**CCND3**	Cyclin D3	↑	NM_001136017.2	Hs.534307
	**FAM83D**	Family with sequence similarity 83, member D	↑	NM_030919.2	Hs.726442
	**MCM7**	Minichromosome maintenance complex component 7	↑	NM_005916.3	Hs.438720
	**NCAPG**	Non-SMC condensin I complex, subunit G	↑	NM_022346.4	Hs.567567
	**PA2G4**	Proliferation-associated 2G4, 38kDa	↑	NM_006191.2	Hs.524498
	**PSMD14**	Proteasome (prosome, macropain) 26S subunit, non-ATPase, 14	↑	NM_005805.5	Hs.740477
	**PSMD2**	Proteasome (prosome, macropain) 26S subunit, non-ATPase, 2	↑	NM_002808.3	Hs.518464
	**SNHG3-RCC1**	Regulator of chromosome condensation 1	↑	NM_001048194.2	Hs.469723
	**THBS1**	Thrombospondin 1	↑	NM_003246.2	Hs.164226

	**TFDP1**	Transcription factor Dp-1	↑	NM_007111.4	Hs.79353
	**UHRF1**	Ubiquitin-like with PHD and ring finger domains 1	↑	NM_001048201.1	Hs.108106
	**CDK4**	Cyclin-dependent kinase 4	↓	NM_000075.3	Hs.95577
	**CDKN1B**	Cyclin-dependent kinase inhibitor 1B (p27, Kip1)	↓	NM_004064.3	Hs.238990
	**DUSP1**	Dual specificity phosphatase 1	↓	NM_004417.3	Hs.171695
	**HEPACAM**	Hepatic and glial cell adhesion molecule	↓	NM_152722.4	Hs.745294
	**MAPK6**	Mitogen-activated protein kinase 6	↓	NM_002748.3	Hs.411847
	**PIM1**	Pim-1 oncogene	↓	NM_002648.3	Hs.81170
	**MCM4**	Minichromosome maintenance complex component 4	↑	NM_005914.3	Hs.460184
	**RPRC1**	MAP7 domain containing 1	↑	NM_018067.3	Hs.356096
	**CCNB1**	Cyclin B1	↑	NM_031966.3	Hs.23960
	**MCM2**	Minichromosome maintenance complex component 2	↑	NM_004526.3	Hs.477481
	**MCM6**	Minichromosome maintenance complex component 6	↑	NM_005915.5	Hs.444118
	**MCM3**	Minichromosome maintenance complex component 3	↑	NM_002388.4	Hs.179565
	**CDKN1A**	Cyclin-dependent kinase inhibitor 1A (p21, Cip1)	↑	NM_000389.4	Hs.370771
	**CCNB2**	Cyclin B2	↑	NM_004701.3	Hs.194698
proliferation	**TIMP3**	TIMP metallopeptidase inhibitor 3	↓	NM_000362.4	Hs.644633
	**APOA5**	Apolipoprotein A-V	↓	NM_052968.4	Hs.283923
	**G6PC**	Glucose-6-phosphatase, catalytic subunit	↓	NM_000151.3	Hs.212293
	**HPN**	Hepsin	↓	NM_182983.2	Hs.182385
	**SERPINE1**	Serpin peptidase inhibitor, clade E (nexin, plasminogen activator inhibitor type 1), member 1	↓	NM_000602.4	Hs.414795
	**ANG**	Angiogenin, ribonuclease, RNase A family, 5	↓	NM_001145.4	Hs.283749
	**CDK4**	Cyclin-dependent kinase 4	↓	NM_000075.3	Hs.95577
	**CDKN1B**	Cyclin-dependent kinase inhibitor 1B (p27, Kip1)	↓	NM_004064.3	Hs.238990
	**HIF1A**	Hypoxia inducible factor 1, alpha subunit (basic helix-loop-helix transcription factor)	↓	NM_001530.3	Hs.597216
	**JUN**	Jun B proto-oncogene	↓	NM_002229.2	Hs.25292
	**NRAS**	Neuroblastoma RAS viral (v-ras) oncogene homolog	↓	NM_002524.4	Hs.486502
	**TGM2**	Transglutaminase 2 (C polypeptide, protein-glutamine-gamma-glutamyltransferase)	↓	NM_004613.2	Hs.517033
	**VEGFA**	Vascular endothelial growth factor A	↓	NM_001025366.2	Hs.73793

	**SERPINA1**	Serpin peptidase inhibitor, clade A (alpha-1 antiproteinase, antitrypsin), member 1	↓	NM_000295.4	Hs.525557
	**PIM1**	Pim-1 oncogene	↓	NM_002648.3	Hs.81170
	**TNFSF10**	Tumor necrosis factor (ligand) superfamily, member 10	↓	NM_003810.3	Hs.478275
	**ABCB7**	ATP-binding cassette, sub-family B (MDR/TAP), member 7	↓	NM_004299.4	Hs.370480
	**BTG1**	B-cell translocation gene 1, anti-proliferative	↓	NM_001731.2	Hs.255935
	**IGF2**	Insulin-like growth factor 2 (somatomedin A)	↓	NM_000207.2	Hs.272259
	**ADA**	Adenosine deaminase	↓	NM_000022.2	Hs.654536
	**MDK**	Midkine (neurite growth-promoting factor 2)	↓	NM_001012334.2	Hs.82045
angiogenesis	**ANG**	Angiogenin, ribonuclease, RNase A family, 5	↓	NM_001145.4	Hs.283749
	**ANXA2**	Annexin A2	↓	NM_001002858.2	Hs.511605
	**CDH2**	Cadherin 2, type 1, N-cadherin (neuronal)	↓	NM_001792.3	Hs.464829
	**CTGF**	connective tissue growth factor	↓	NM_001901.2	Hs.410037
	**HIF1A**	Hypoxia inducible factor 1, alpha subunit (basic helix-loop-helix transcription factor)	↓	NM_001530.3	Hs.597216
	**JUN**	Jun B proto-oncogene	↓	NM_002229.2	Hs.25292
	**LOX**	Lysyl oxidase	↓	NM_002317.5	Hs.102267
	**TGM2**	Transglutaminase 2 (C polypeptide, protein-glutamine-gamma-glutamyltransferase)	↓	NM_004613.2	Hs.517033
	**VEGFA**	Vascular endothelial growth factor A	↓	NM_001025366.2	Hs.73793
	**SERPINE1**	Serpin peptidase inhibitor, clade E (nexin, plasminogen activator inhibitor type 1), member 1	↓	NM_000602.4	Hs.414795
	**ANGPT1**	Angiopoietin 1	↓	NM_001146.3	Hs.369675
	**PLG**	Plasminogen	↓	NM_000301.3	Hs.143436
	**NRP1**	Neuropilin 1	↓	NM_003873.5	Hs.131704
	**RAMP2**	Receptor (G protein-coupled) activity modifying protein 2	↓	NM_005854.2	Hs.514193
	**SDC4**	Syndecan 4	↓	NM_002999.3	Hs.632267
cell adhesion	**FREM1**	FRAS1 related extracellular matrix 1	↓	NM_144966.5	Hs.50850
	**CDH2**	Cadherin 2, type 1, N-cadherin (neuronal)	↓	NM_001792.3	Hs.464829
	**CADM1**	Cell adhesion molecule 1	↓	NM_014333.3	Hs.370510
	**COL7A1**	collagen, type VII, alpha 1	↓	NM_000094.3	Hs.476218

**CTGF**	connective tissue growth factor	↓	NM_001901.2	Hs.410037
**HEPACAM**	Hepatic and glial cell adhesion molecule	↓	NM_152722.4	Hs.745294
**IGFBP7**	Insulin-like growth factor binding protein 7	↓	NM_001553.2	Hs.479808
**ITGB2**	integrin, beta 2 (complement component 3 receptor 3 and 4 subunit)	↓	NM_000211.3	Hs.375957
**SPP1**	secreted phosphoprotein 1	↓	NM_001040058.1	Hs.313
**MMP11**	matrix metallopeptidase 11 (stromelysin 3)	↓	NM_005940.3	Hs.143751
**TIMP3**	TIMP metallopeptidase inhibitor 3	↓	NM_000362.4	Hs.644633
**TFPI**	Tissue factor pathway inhibitor (lipoprotein-associated coagulation inhibitor)	↓	NM_006287.4	Hs.516578
**COL6A1**	Collagen, type VI, alpha 1	↓	NM_001848.2	Hs.474053
**F5**	Coagulation factor V (proaccelerin, labile factor)	↓	NM_000130.4	Hs.30054
**TIMP2**	TIMP metallopeptidase inhibitor 2	↓	NM_003255.4	Hs.633514
**FGG**	fibrinogen gamma chain	↓	NM_000509.4	Hs.727584
**VTN**	vitronectin	↓	NM_000638.3	Hs.2257
**FN1**	Fibronectin 1	↓	NM_212482.1	Hs.203717
**F7**	coagulation factor VII (serum prothrombin conversion accelerator)	↓	NM_000131.4	Hs.36989

*gene expression highlighted in bold font showing at least 1.5-fold change in HepG2_PBLD groups compared with that in the control groups.($P<1x10^{-3}$)

Table Legend: Microarray analysis showed that overexpression of PBLD resulted in inactivation of several tumorigenesis-related signaling pathways, including MAPK, NF-κB, EMT, angiogenesis and others listed in detail above.
